# Cold Tolerance Regulated by the Pyruvate Metabolism in *Vibrio parahaemolyticus*

**DOI:** 10.3389/fmicb.2019.00178

**Published:** 2019-02-06

**Authors:** Tengfei Xie, Rui Pang, Qingping Wu, Juemei Zhang, Tao Lei, Yanping Li, Juan Wang, Yu Ding, Moutong Chen, Jianlin Bai

**Affiliations:** ^1^Guangdong Institute of Microbiology, State Key Laboratory of Applied Microbiology Southern China, Guangdong Provincial Key Laboratory of Microbial Culture Collection and Application, Guangdong Open Laboratory of Applied Microbiology, Guangzhou, China; ^2^College of Food Science, South China Agricultural University, Guangzhou, China; ^3^Department of Food Science & Technology, Jinan University, Guangzhou, China

**Keywords:** *Vibrio parahaemolyticus*, cold, pyruvate, transcriptome, proteome

## Abstract

*Vibrio parahaemolyticus* is a common foodborne pathogen found in seafood, and represents a major threat to human health worldwide. Low-temperature storage is an important seafood processing method, but is not sufficient to completely eliminate the bacteria and avoid foodborne illness. To determine the mechanisms behind such cold tolerance, RNA-seq and iTRAQ analyses were first performed to obtain the global transcriptomic and proteomic patterns of frozen squid and clinical *V. parahaemolyticus* isolates under cold conditions. The integrated analysis revealed the modulation of multiple pathways such as the co-occurrence of down-regulated pyruvate metabolism and up-regulated fatty acid biosynthesis, which likely contribute to *V. parahaemolyticus* cold tolerance. Furthermore, we found that increasing concentrations of pyruvate can reduce the fatty acid content to influence *V. parahaemolyticus* growth in cold conditions. Thus, regulation of pyruvate concentration may be an effective method to control this seafood-borne pathogen.

## Introduction

The causative agent of diarrheal disease, *Vibrio parahaemolyticus*, is the major pathogen responsible for seafood associated gastroenteritis in humans worldwide (Samoǐlenko et al., 2003). Seafood is very popular in China, and its higher consumption is correlated with an increase in the overall living standard of the population. However, this high rate of seafood consumption is also accompanied by high rates of foodborne illness. In the city of Sanya, China alone, there were 29 outbreaks caused by *V. parahaemolyticus* resulting in 499 illnesses from 2010 to 2016, accounting for about half of all cases of microbiological food poisoning (Deng et al., 2017). Raw seafood is generally subject to low-temperature storage as an important food preservation method, which can effectively limit bacterial growth and metabolism (Graumann and Marahiel, 1996). However, *V. parahaemolyticus* has still been isolated from seafood that has gone through the low-temperature treatment to ultimately cause foodborne illness (Wang et al., 2016) suggesting a specific mechanism of cold tolerance that has yet to be elucidated.

Previous studies have indicated various adaptations of microorganisms to low temperature, including reduced affinity of enzymes for their substrates, decreased thermal energy and reaction rates, and increased aqueous viscosity (D’Amico et al., 2006). Complex cold shock responses, including cold shock protein (CSP) production, DNA supercoiling modifications, and membrane fluidity maintenance, can help a bacterium survive under detrimental cold conditions (Horn et al., 2007). Nevertheless, different microorganisms have evolved unique strategies in response to low temperatures. For example, unsaturated fatty acids were shown to be effective cold-tolerant factors in *Pseudomonas* sp. (Garba et al., 2016). Another four proteins (ATP-dependent, ClpP, pyruvate kinase and a putative glycoprotein endopeptidase) appear to play important roles in cold adaptation for *Lactobacillus acidophilus*. Despite this basic information on bacterial cold adaptation, the complete strategies, pathways, and signals that compensate for low-temperature metabolism in *V. parahaemolyticus* remain poorly understood (Amato and Christner, 2009).

High-throughput sequencing technology has now made it possible to obtain detailed transcriptomic profiles and improve our understanding of the genetic variation involved in pathogen infection and virulence (Jiang et al., 2016; Sun et al., 2016). The proteome, a term coined by Wilkins in 1992, refers to all of the proteins expressed by the genome, which includes those assessed at the cellular or organismal level (Wasinger et al., 1995). Isobaric tags for relative and absolute quantitation (iTRAQ) is a relatively advanced quantitative proteomic technique that enables relative protein measurements simultaneously with determination of the absolute levels of a target protein using synthetic isobaric peptide standards (Ross et al., 2004), To date, analysis of the *V. parahaemolyticus* proteome has mostly been based on the results of sodium dodecyl sulfate (SDS)-polyacrylamide gel electrophoresis and two-dimensional electrophoresis methods (Chen et al., 2016), but a comprehensive understanding of the components involved in the activation, regulation, and tolerance of low-temperature remains incomplete. The recent advent of sequencing technologies has now made it possible to obtain detailed transcriptomic and proteome profiles of this pathogen in different environments. Indeed, microarray analysis helped to elucidate the effects of salt and acid stress on the *V. parahaemolyticus* transcriptome (Yang et al., 2010; Sun et al., 2014).

To further illustrate the molecular mechanisms driving the low-temperature adaptation of *V. parahaemolyticus*, we employed RNA-sequencing (RNA-seq) and iTRAQ methods to obtain the global transcriptome and proteome patterns of *V. parahaemolyticus* under low-temperature, respectively. Analysis of these transcriptional and proteome profiles is expected to broaden our understanding of the pathways induced in *V. parahaemolyticus* to facilitate survival under low-temperature, which can help to identify potential targets for developing food processing strategies to achieve more effective control of this pathogen and prevent widespread outbreaks of foodborne illness.

## Materials and Methods

### Culture and Incubation Conditions

*Vibrio parahaemolyticus* strains V82 (isolated from frozen squid) and VL8 (clinical isolate, Xie et al., 2017) were grown overnight at 37°C in tryptic soytone broth (TSB) medium (HuanKai Microbial, Guangzhou, China). Dilutions of each culture were prepared up to 1:10^3^ dilution (TSB medium), further incubated at 37°C for 12 h, and then transferred to 4°C for 12 h. The control group was cultured at 37°C for 24 h continuously. Growth status was measured spectrophotometrically based on the optical density at 600 nm. According to the growth curve, stable phase strains were selected for RNA-seq and iTRAQ analysis (Supplementary Figure S1).

### RNA-Seq Analysis

The total RNA was extracted using Bacterial RNA Kit (Omega Bio-Tek, Salt Lake City, UT, United States) according to the manufacturer’s instructions. The complementary DNA (cDNA) libraries from two replicates of all samples were constructed and sequenced by GENE DENOVO, Ltd. (Guangzhou, China) on the Illumina sequencing platform (HiSeq^TM^ 2500), producing 150-bp single-end reads. All clean reads were aligned to the reference genome (GCF_001558495.1) of *V. parahaemolyticus* using Tophat2, and the fragments per kilobases per million values for the genes were estimated by Cufflinks software (Trapnell et al., 2012). Based on the threshold of log_2_ fold change ≥ 1 and false discovery rate (FDR) ≤ 0.05, the differentially expressed genes (DEGs) in each comparison were determined by the Cuffdiff module. All DEGs were annotated to the Gene Ontology database^1^ and the Kyoto Encyclopedia of Genes and Genomes (KEGG) pathway database (Kanehisa et al., 2008). The functional enrichment was determined by comparison to the annotation of reference transcripts (FDR ≤ 0.05). To support this analysis, 20 DEGs were randomly selected for measurement of expression levels in each sample by quantitative reverse transcription-polymerase chain reaction (qRT-PCR).

### iTRAQ Analysis

To enrich the total proteins, three biological replicates of all samples were separately ground into powder in liquid nitrogen, homogenized in 1 mL lysis buffer (10% w/v SDS, 0.1 M dithiothreitol in 0.1 M Tris-HCl, pH 7.6). Then sonication was in an ice bath for 15 cycles (work 5 s, stop 2 s) at 35 kHz. After super-centrifugation (30000 *g*, 4°C for 15 min), the supernatant was precipitated in 10% trichloroacetic acid /acetone, centrifuged at 30,000 *g*, 15 min at 4°C. Three acetone washes were performed on the protein precipitate. Protein precipitates were dissolved in the lysis buffer, protein concentration was measured by Bradford assay protein assay kit (Bio-Rad, United States) and 100 μg aliquots of each sample were used for proteomic experiments. The Filter-aided sample preparation-method (FASP) by Wiśniewski et al. (2009) that, employing ultrafiltration devices, allows to perform SDS removal, buffer exchange, chemical modification, and protein digestion to obtain peptides mixtures suitable for mass-spectrometric analysis was used to prepare peptide samples for iTRAQ experiments. Trypsin digestion (enzyme to protein ratio 1:50) was carried out at 37°C for 4 h. 4-plex iTRAQ labeling experiments were performed according to the manual provided by AB SCIEX, Pte. Ltd. (Redwood City, CA, United States) (Ross et al., 2004). Strong cation exchange were conducted in a Gemini-NX 5u C18 110A 150 mm × 4.6 mm column (Phenomenex, Guangzhou, China) by using a LC-20AB HPLC Pump system (Shimadzu, Japan). The process is as follows: uv wavelength: 214 nm; flow rate, 1000 μL/min; washing-gradient of liquid chromatography: Time(min), B%: 1, 5%; 18, 30%; 20, 80%; 24, 80%; 24.1, 5%; 30, stop. Reversed-phase liquid chromatography-tandem mass spectrometry were performed by SAGENE, Co., Ltd. (Guangzhou, China) in C18 enriching column (3 μm, ID 100 μm, 20 mm length) and separation column (1.9 μm, ID75 μm, 100 mm length) by using a Triple TOF 6600 (Applied Biosystems, United States). The process is as follows: flow rate, 300 nL/min; washing-gradient of liquid chromatography: Time(min), B%: 0.1, 8%; 55, 25%; 65, 80%; 70, 80%; 70.1, 2%; 75, 2%; stop. The peptides were identified by ProteinPilot 5.0 (AB Sciex) and matched to the reference transcripts of protein sequences of *V. parahaemolyticus* in SwissProt/UniProt database (identification parameters are shown in the Supplementary Table S1). The statistically significant differentially-accumulated proteins between treatment groups were determined based on the threshold of protein abundance ratio ≥ 1.5 (up-accumulation) or ≤ 0.667 (down-accumulation) (*p*-value ≤ 0.05, *t*-test). The protein–protein interaction network of differentially-accumulated proteins was analyzed using STRING (Szklarczyk et al., 2017). KEGG pathway annotation was performed as above description, and pathway overlaps with DEGs were identified with a custom Perl script.

### Quantitative Real-Time PCR (qRT-PCR) Analysis

The total RNA was extracted using Bacterial RNA Kit (Omega Bio-Tek) according to the manufacturer’s instructions. RNA degradation and contamination were monitored on 1% agarose gels and the RNA concentration was quantified cDNA was synthesized using M-MLV First Strand cDNA Synthesis Kit (Omega Bio-Tek) and was quantified using Perfectstart SYBR Green qPCR master mix (Omega Bio-Tek). qRT-PCR was performed on the QuanStudio^TM^ Real-Time PCR system (Applied Biosystems, Foster City, CA, United States). The mRNA level of each gene was normalized to that of 16S rRNA. A list of all primers used in this study is provided in Supplementary Table S2.

### Fatty Acids Detection

*Vibrio parahaemolyticus* strain V82 was grown in TSB at 37°C, and then transferred to 4°C for the low-temperature treatment. The cultures were harvested by centrifugation (5000 × *g*, 4°C) and the fatty acid compositions were determined as described by Zhu et al. (2005) and the National Food Safety Standards of China guideline GB5009.168-2016 for examination of fatty acids.

### Detection of *V. parahaemolyticus* by Confocal Laser Scanning Microscopy

Confocal laser scanning microscopy (CLSM) (Zeiss, Berlin, Germany) analysis was performed using a LIVE/DEAD^®^ BacLight^TM^ Bacterial Viability and Counting Kit (Invitrogen, Carlsbad, CA, United States) with an appropriate mixture of the SYTO 9 and propidium iodide (PI).

### Statistical Analysis

Integrated analysis of the transcriptome and proteome data was conducted using locally designed Perl scripts. For the statistical analysis of qRT-PCR, the relative mRNA levels and -ΔΔCT values were calculated from the obtained cycle threshold values of three biological replicates (Livak and Schmittgen, 2012). The results of fatty acids detection are indicated as mean standard error of the mean (*n* = 3), and analyzed using significant range tests. Differences between groups were considered significant at *P* < 0.05.

## Results

### Transcriptome Profiles for Strains VL8 and V82 Under Cold Stress

**FIGURE 1:**
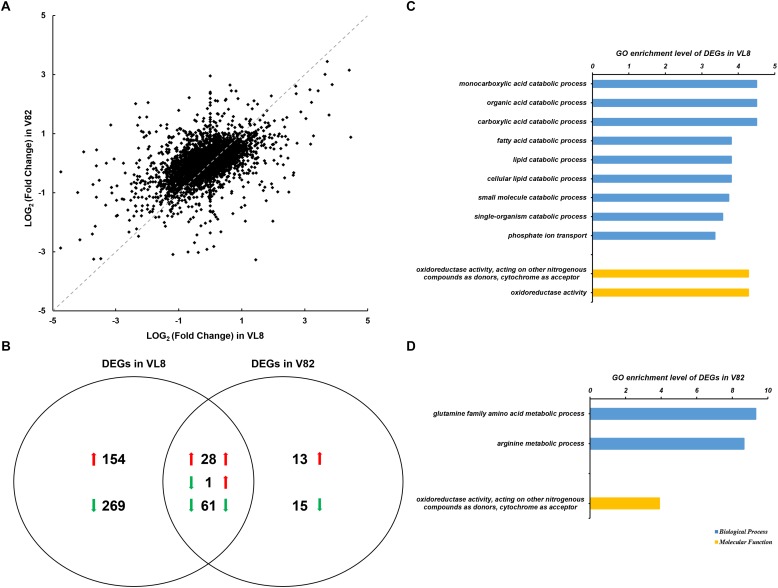
Analyses of the RNA-seq data of two *Vibrio parahaemolyticus* strains under cold stress. The comparison was performed on the 4°C (supplemented) to 37°C (control) in each strain. **(A)** Scatter plots of log2 (Fold Change) per gene from the comparisons of two strains. **(B)** Venn diagrams of differentially expressed genes (DEGs) from the comparisons of two strains. GO enrichment analysis of DEGs from the comparison of VL8 **(C)** and V82 **(D)**, the GO annotation of whole transcripts of *V. parahaemolyticus* was used as background.

RNA-seq analysis was conducted to determine global changes at the transcript level for *V. parahaemolyticus* strains VL8 and V82 under cold stress, which revealed numerous DEGs for both strains cultured at 4 and 37°C. There was also obvious variation in *V. parahaemolyticus* gene expression profiles between the pathogenic strain VL8 and environmental strain V82 (Figure 1A, *R*^2^ = 0.2498). The cold stress elicited 513 DEGs in VL8, representing approximately 11.01% of all genes in *V. parahaemolyticus*. Of these, 182 genes were significantly up-regulated and 331 genes were significantly down-regulated (Figure 1B and Supplementary Table S3). The DEGs in V82 were enriched in functional categories of multiple catabolic processes, phosphate ion transport, and oxidoreductase activity (Figure 1C and Supplementary Table S4).

In contrast, 118 genes, which accounted for only 2.53% of all genes in the bacterium, showed significantly differential expression in V82, including 42 up-regulated and 76 down-regulated genes that were enriched in the functional categories of glutamine and arginine metabolic processes, and oxidoreductase activity (Figure 1D). Interestingly, most of the DEGs in V82 overlapped with those in VL8 (66.67% of up-regulated genes and 80.26% of down-regulated genes, Figure 1B), reflecting that most of the responses to cold stress in V82 should be similar to those in VL8.

**FIGURE 2:**
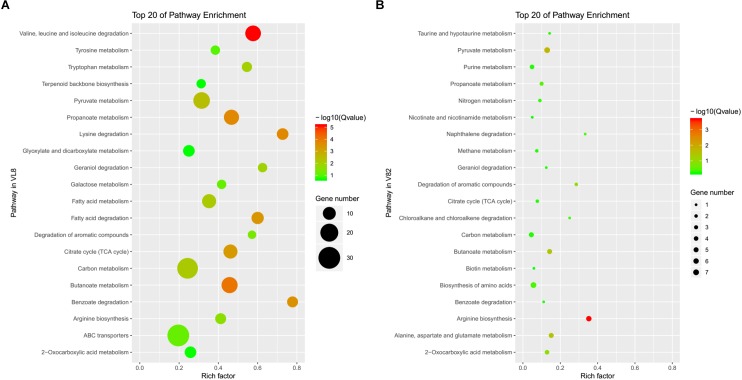
The top 20 of enriched pathways annotated in the KEGG data library of DEGs from the comparisons of strains VL8 **(A)** and V82 **(B)**.

The annotation of KEGG pathways also demonstrated the differences and similarities between the responses of strains VL8 and V82 to cold stress. There were more DEGs annotated to the pathway of the ATP-binding cassette (ABC) transporter in VL8 (Figure 2A), while more DEGs in V82 were annotated to the pathway of the biosynthesis of amino acids (Figure 2B). In addition, the DEGs identified in these two strains were commonly enriched in the pathways of carbon metabolism, arginine biosynthesis, pyruvate metabolism, and butanoate metabolism.

Moreover, the randomly selected DEGs showed significantly differential expression levels by qRT-PCR analysis that were consistent with the results of RNA-seq analysis, suggesting the reliability of the RNA-seq results (Supplementary Figure S2).

### Global Changes at the Protein Levels in VL8 and V82 Under the Cold Stress and Their Correlation With Transcript Levels

**FIGURE 3:**
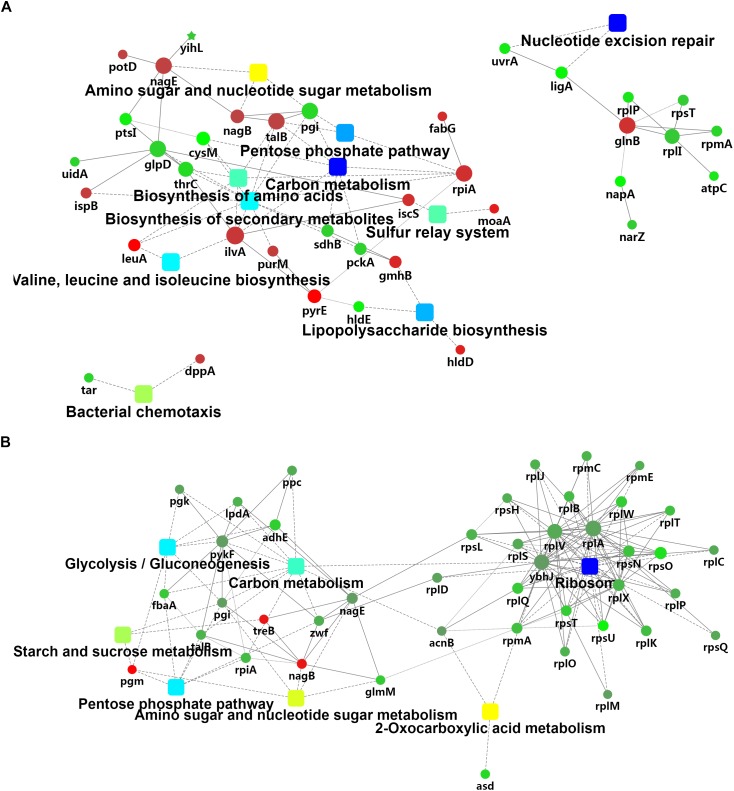
Analyses of the iTRAQ data of two *V. parahaemolyticus* strains under cold stress. The comparison was performed on the 4°C (supplemented) to 37°C (control) in each strain. A network analysis was performed on these differentially-accumulated proteins from the comparison of VL8 **(A)** and V82 **(B)**.

The iTRAQ mass spectrometric analysis was performed on three replicates of the cold-treated VL8 and V82 experiments to identify the differences in protein levels. In total, 1539 proteins were verified with quantitative information in all three replicates. The cold stress resulted in 20 up-accumulated and 25 down-accumulated proteins in VL8, and 17 up-accumulated and 129 down- accumulated proteins in V82 (Supplementary Tables S5, S6). Cold stress induced distinct changes in carbon metabolism and the pentose phosphate pathway for both strains (Figure 3A,B). We also observed changes in protein clusters of nucleotide excision repair (Figure 3A) and ribosome (Figure 3B), which might indicate a response to prevent the misfolding of proteins under cold stress.

**FIGURE 4:**
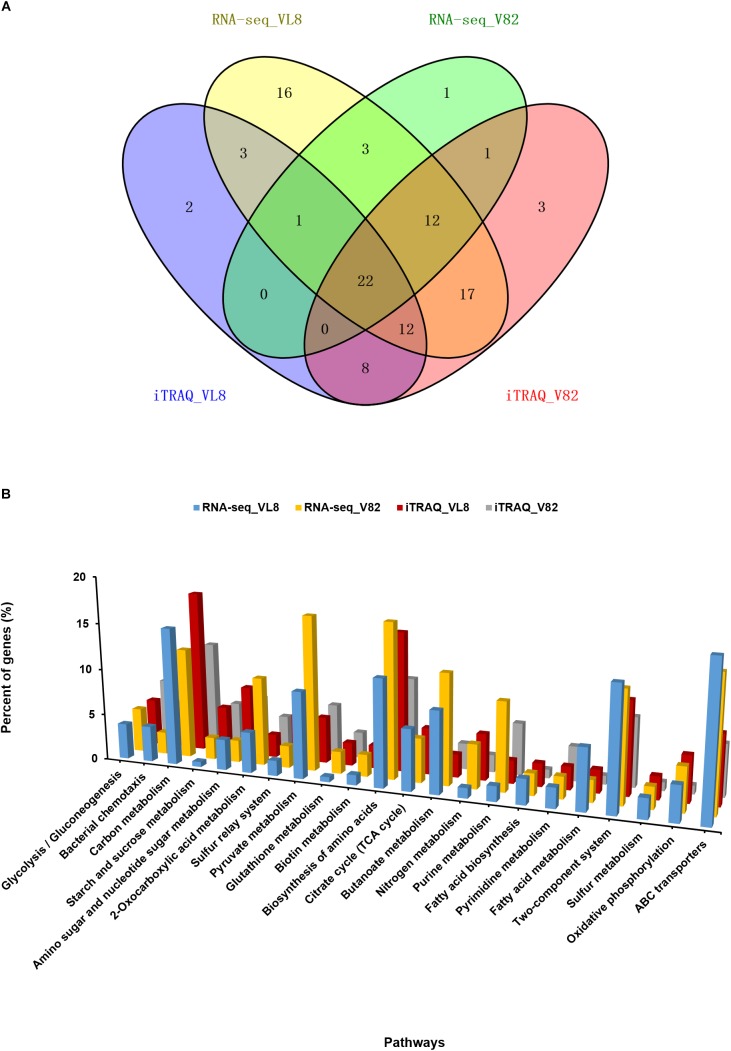
Combined analyses of the RNA-seq and iTRAQ data of two *V. parahaemolyticus* strains under cold stress. **(A)** Venn diagrams of regulated pathways from RNA-seq and iTRAQ analyses from the comparison of strains VL8 and V82. **(B)** Details of 22 overlapping pathways identified in all RNA-seq and iTRAQ datasets. The Y-axis represented the percent of genes in all DEGs or differentially expressed proteins.

We then performed an integrated analysis of the iTRAQ and RNA-seq data sets. Overall, 38 and 42 overlapping pathways were found between the RNA-seq and iTRAQ data in VL8 and V82, respectively (Figure 4A). Moreover, a total of 22 pathways were found to be affected in all data sets, including some important metabolic pathways such as carbon metabolism, biosynthesis of amino acids, and fatty acid metabolism (Figure 4B and Supplementary Table S7). Interestingly, the numbers of affected genes were relatively high in the pathways of pyruvate metabolism and ABC transporters, reflecting their important roles in the acclimation to cold of *V. parahaemolyticus*.

### Decrease in Pyruvate Metabolism Confers Resistance to Cold Stress in *V. parahaemolyticus*

**FIGURE 5:**
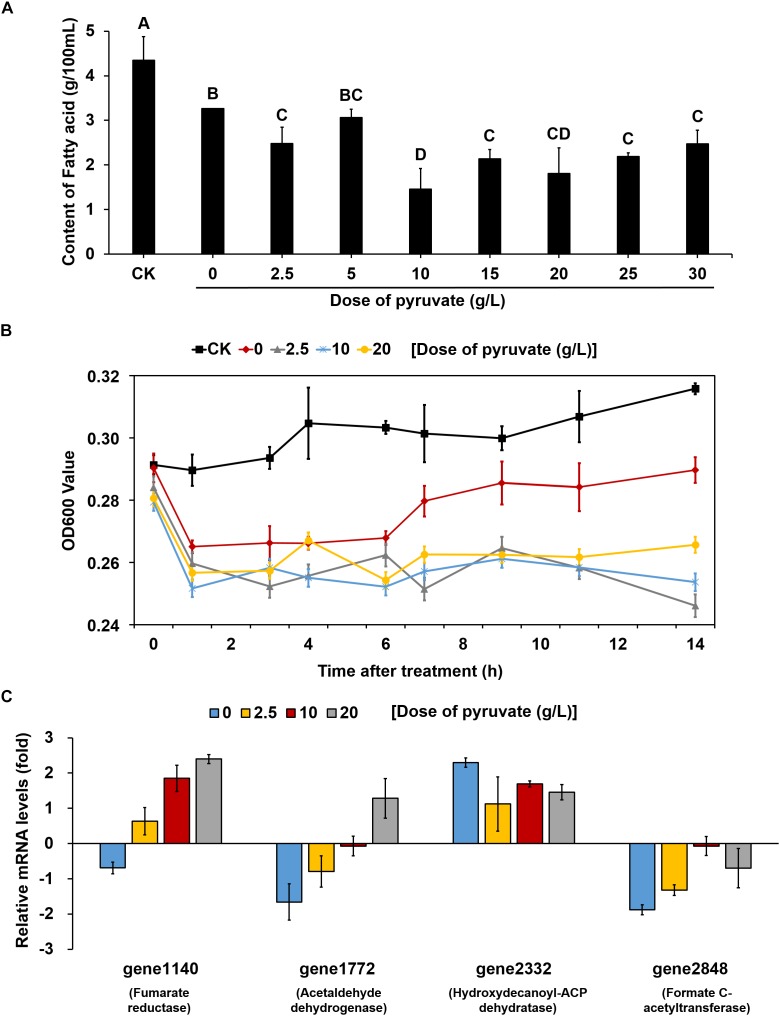
The supplement of exogenous pyruvate reduced the accumulation of fatty acid and effect on *V. parahaemolyticus* growth at low temperature by reversing the expression patterns of key genes. **(A)** Changes in the content of total fatty acids in unit of bacteria supplemented with different doses of pyruvate at 4°C. CK represents bacteria grown at 37°C without any treatment. All bacteria were detected in the same OD value. The values sharing the same letter are not significantly different at *P* < 0.05 (least significant range tests). **(B)** Changes in the growth of bacteria supplemented with different doses of pyruvate at 4°C. CK represents bacteria grown at 37°C without any treatment. **(C)** Changes in the mRNA levels of key genes within pyruvate metabolism and fatty acid biosynthesis pathways of bacteria supplemented with different doses of pyruvate at 4°C. All data are normalized relative to the mRNA levels of bacteria grown at 37°C without any treatment. All values are mean of three biological replicates.

Consistent with the significant change detected in the pyruvate metabolic pathway in both VL8 and V82 under cold stress based on the integrated analysis, the KEGG mapping results demonstrated that the biosynthesis and utilization of pyruvate were significantly decreased (Supplementary Figure S3). Consequently, tricarboxylic acid (TCA) cycle pathway was also down-regulated (Figure 5B). Interestingly, the lower metabolism of pyruvate led to the promotion of fatty acid biosynthesis, resulting in suppression of fatty acid degradation (Supplementary Figure S3).

To clarify whether the up-regulation of fatty acid biosynthesis was caused by a decrease in pyruvate metabolism, different concentrations of pyruvate were added to the culture medium inoculated and maintained at 4°C. Compared with the cultures without pyruvate supplement, the fatty acid contents in almost all pyruvate supplement cultures (except for those supplemented with 5 g/L pyruvate) were significantly reduced (Figure 5A). We then measured the growth curves of cultures supplemented with three concentrations of pyruvate (2.5, 10, and 20 g/L) at 4°C, which were compared to those of cultures without pyruvate supplement at both 4 and 37°C. At physiological temperature (37°C), the bacteria grew gradually whereas growth was markedly suppressed at low temperature (4°C) (Figure 5B). The cultures without pyruvate supplement could overcome the cold stress over time and slowly resumed normal growth. However, the pyruvate-supplemented populations could barely recover growth throughout the experiment.

**FIGURE 6:**
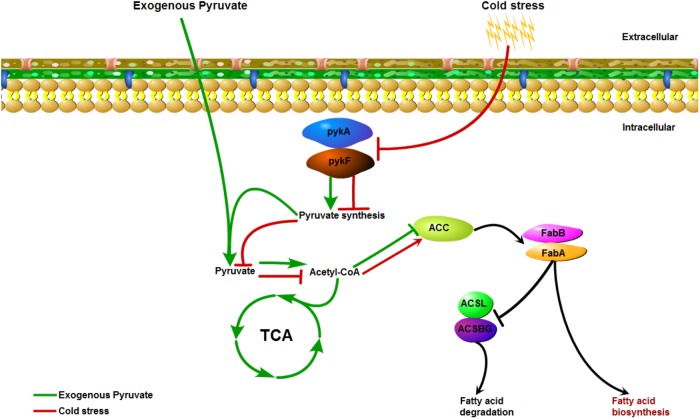
The possible action mode mediated by pyruvate metabolism that underlie low-temperature adaptation of *V. parahaemolyticus*.

Quantitative reverse transcription-polymerase chain reaction analysis was conducted to further determine the changes in the expression of key genes involved in the corresponding pathways identified to be enriched under cold stress. Three genes in the pyruvate metabolism pathway, gene1140 (fumarate reductase), gene1772 (acetaldehyde dehydrogenase), and gene2848 (formate *C*-acetyltransferase), were significantly down-regulated under 4°C (Figure 5C). Notably, the OD600 values decreased for cultures at low temperature during the first 1 h. Confocal laser scanning microscopy images indicated that most bacteria died at 4°C compared to the culture at 37°C (Supplementary Figure S4). Thus, we inferred that the decrease in OD600 values were mainly attributed to cellular lysis of dead bacterial. However, the addition of pyruvate disrupted the down-regulation of these genes, and even elevated their mRNA levels. Similarly, the up-regulation of a key gene (gene2332, hydroxydecanoyl-ACP dehydratase) in the fatty acid biosynthesis pathway was also prevented to a certain degree by the pyruvate treatment. Collectively, these results suggested that cold stress induced the accumulation of fatty acids by reducing pyruvate metabolism, but that supplementation of exogenous pyruvate would disrupt this process (Figure 6).

## Discussion

In parallel with improvements in the standard of life, consumers are increasingly concerned with both the taste and nutritive value of their food. To meet this consumer demand, food producers must continuously adjust processing methods and storage conditions to maintain the original taste and quality of foods (Ma, 2016). Post-harvest processing is particularly important for maintaining the quality and taste of seafood, and low-temperature storage is an essential aspect of this process so as to challenge the survival of *V. parahaemolyticus* given that its virulence decreases in a low-temperature environment, including a reduction in hemolysin and protease production to induce a cytotoxic effect (Mahoney et al., 2010). Indeed, microorganism fitness (growth and survival) has been shown to be significantly linked with temperature (Boonyawantang et al., 2012). Thus, to survive and adapt to changes of the outside environment, microorganisms including foodborne pathogens can undergo a series of changes in gene regulation to control relevant metabolic pathways to combat the negative effects of stress conditions (Azizoglu et al., 2009). Given that *V. parahaemolyticus* can clearly survive under the low-temperature storage conditions in seafood and cause foodborne illness, we assessed the global changes in transcript and protein levels of a clinical and a frozen food *V. parahaemolyticus* isolate at low temperature to determine the adaptation mechanism. This integrated analysis revealed several stimulus-response pathways that were induced under cold stress in both strains, including the commonly affected pathways of ABC transporters, pyruvate metabolism, and fatty acid biosynthesis. Although common patterns were detected in both isolates, there were many more unique DEGs identified in VL8, suggesting that the gene expression profile in the environmental strain was relatively more stable under cold stress, while the pathogenic strain would need to mobilize more gene resources to protect itself from the cold stress.

The ABC transporter proteins, also known as highly conserved in ATP-binding sites proteins (Higgins et al., 1986), are found in all domains of life, and are mainly involved in the uptake of nutrients and micronutrients, extrusion of building blocks, and drug resistance by exporting certain toxic substances outside of the cell (Cuthbertson et al., 2010; Locher, 2016). Similar to the present findings, ABC transporters were found to be significantly inhibited in *V. parahaemolyticus* isolates with a mutation in the CSP gene *cspA* to compensate for the deficiency and protect the bacterium from low temperature induced damage (Zhu et al., 2017). Another report showed that *V. parahaemolyticus* ABC transporters demonstrated notable variation in response to acid and toxicity stress (Nydam et al., 2014; Sun et al., 2014). Thus, our results confirm an important role of ABC transporter pathways in response to cold stress at both the transcriptome and proteome levels in *V. parahaemolyticus*, which have been shown to play an important role in low-temperature adaptation of many other bacteria (Allen et al., 2009).

Interestingly, the down-regulation of pyruvate metabolism and the up-regulation of fatty acid biosynthesis co-occurred in this bacterium under cold stress. Fatty acids are crucial metabolites that are present in almost all living organisms, which play essential roles in maintaining membrane integrity with a direct effect on some cellular processes such as environmental adaptation, cellular differentiation, DNA replication, and cell death (Mansilla et al., 2008; Garba et al., 2016). The accumulation of fatty acids has also been considered to confer an advantage to bacteria for growth under low temperature (Suutari and Laakso, 1994), and Mansilla et al. (2004) showed that bacteria could stringently control the production and modulation of fatty acids within varying temperatures to maintain membrane lipids in the correct physical state. Thus, changing the fatty acid composition is a common strategy for bacteria to overcome low-temperature pressure. For example, different kinds of fatty acids were shown to respond to cold stress in *Shewanella piezotolerans* strains (Wang et al., 2009), and multiple genes related to fatty acid metabolism were induced under cold exposure over time in a previous study of *V. parahaemolyticus* (Yang et al., 2009). Changes of lipids and lipid-like molecules in *V. parahaemolyticus* at low temperature were also observed at the metabolome level (Feng et al., 2016). Pyruvate, a small molecule that plays a central role in metabolism, is the final product of glycolysis and the starting substrate for the TCA cycle (Fink, 2003), also acting as a potent and effective reactive oxygen species scavenger to protect bacteria from oxidative stress (Karabeyoğlu et al., 2008). Pyruvic acid affects the metabolic rate of fatty acids, which in turn influences the TCA cycle (de Groot et al., 1995; Zhang et al., 2010).

Besides demonstrating a link to pyruvate metabolism and fatty acid biosynthesis in terms of gene and protein expression changes under cold stress, we found that direct supplementation of exogenous pyruvate to the culture medium could reduce the fatty acid content to suppress *V. parahaemolyticus* growth under low temperature. Based on these results, we inferred that reduction of pyruvate synthesis can decrease the energy metabolism in *V. parahaemolyticus* in the face of cold stress. However, the addition of pyruvate may transmit an error signal to activate the TCA cycle and intensify the utilization rate of fatty acids. Since the accumulation of fatty acids is a significant microbial cold tolerance adaptation, The down-regulation of fatty acid synthesis caused by exogenous pyruvate is a strategy that could induce the death of *V. parahaemolyticus* cells growth at low temperature and improve food safety. These findings provide a new method to control the growth of this pathogen on frozen aquatic products during transport by adjusting the concentration of pyruvic acid to decrease the survival rate of *V. parahaemolyticus* and reduce the potential of contamination.

## Conclusion

This study represents the first investigation of foodborne and clinical *V. parahaemolyticus* isolates in response to low temperature using an integrated transcriptome and proteome analysis. We detected distinct global-level gene expression and protein accumulation profiles, revealing several potential key mechanisms for coping with cold stress, such as modulating the pathways of ABC transporters, pyruvate metabolism, and fatty acid biosynthesis. Moreover, pyruvate addition reduced the fatty acid biosynthesis to suppress *V. parahaemolyticus* growth at low temperature. Our results should facilitate further in-depth research of the cold stress resistance of *V. parahaemolyticus* to provide a potential strategy of controlling this seafood-borne pathogen.

## Author Contributions

TX and RP are the common first authors, finish the article experiment, and write the article together. QW give the idea and experiments support. JZ, TL, YL, JW, YD, MC, and JB help to finish the experiment on article.

## Conflict of Interest Statement

The authors declare that the research was conducted in the absence of any commercial or financial relationships that could be construed as a potential conflict of interest.

## Footnotes

1 http://www.geneontology.org/

## Supplementary Material

FIGURE S1 The growth curve of V82, VL8 at different temperature stages.

FIGURE S2 Differentially expressed genes validated by qRT-PCR analysis.

FIGURE S3 The important pathways affected by cold stress in *V. parahaemolyticus*, including pyruvate metabolism pathway **(A)**, citrate cycle (TCA cycle) pathway **(B)**, fatty acid biosynthesis pathway **(C)**, and fatty acid degradation pathway **(D)**. Red indicates that the gene is up-regulated at low temperature, while green indicates that the gene is down-regulated at low temperature.

FIGURE S4 Laser confocal fluorescence microscopy indicating the status of bacteria supplied with different doses of pyruvate at 4°C. **(A)** Initial sample at 37°C without any treatment. **(B)** Sample at 37°C for 1 h. **(C)** Sample without pyruvate supplement at 4°C for 1 h. **(D)** Sample with 2.5 g/L pyruvate supplement at 4°C for 1 h. **(E)** Sample with 10 g/L pyruvate supplement at 4°C for 1 h. **(F)** Sample with 20 g/L pyruvate supplement at 4°C for 1 h. Green cells represent the alive bacteria, while red cells represent the dead bacteria.

TABLE S1 Parameters used for database identification by ProteinPilo.

TABLE S2 The primers used in qRT-PCR.

TABLE S3 Differentially expressed genes in *V. parahaemolyticus* strain VL8 under cold stress.

TABLE S4 Differentially expressed genes in *V. parahaemolyticus* strain V82 under cold stress.

TABLE S5 Differentially accumulated proteins identified from VL8.

TABLE S6 Differentially accumulated proteins identified from V82.

TABLE S7 Details of 22 overlapping pathways identified in all RNA-seq and iTRAQ datasets.
