# Supplementary figures and images for: Cold Tolerance Regulated by the Pyruvate Metabolism in Vibrio parahaemolyticus
Source: Front Microbiol. 2019 Feb 6;10:178. doi: 10.3389/fmicb.2019.00178 (PMC6372572; doi:10.3389/fmicb.2019.00178)

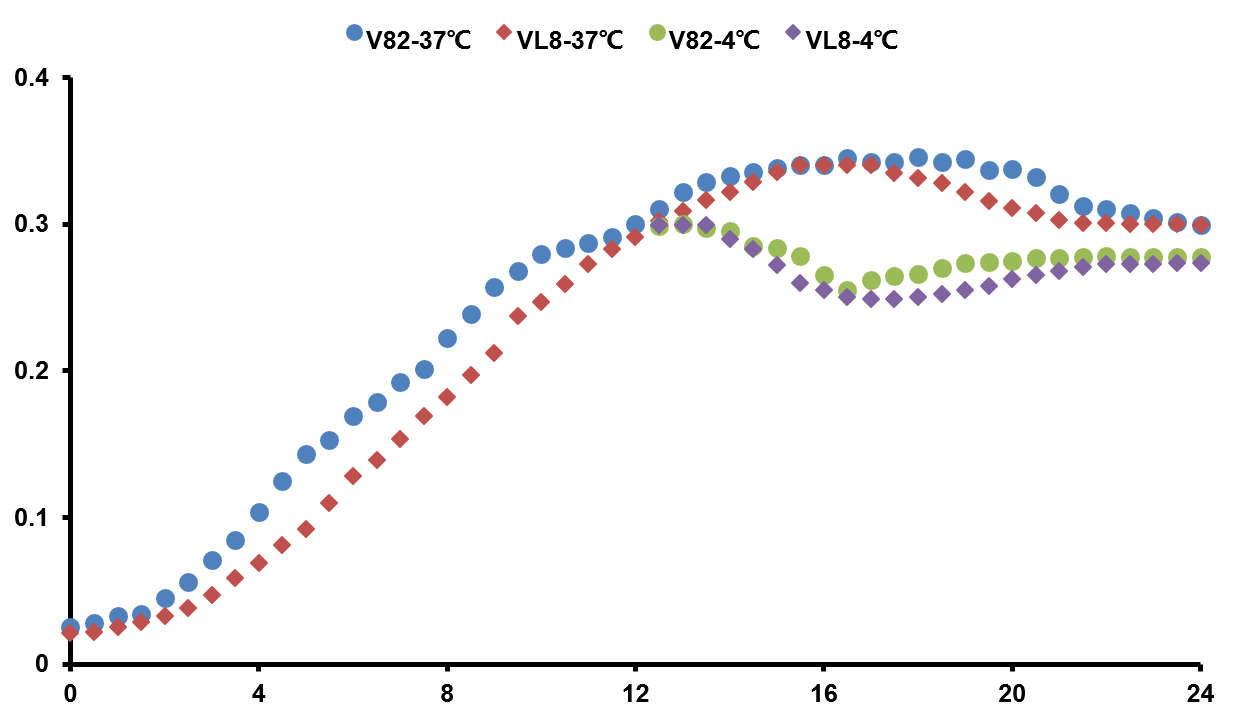

Supplement: FIGURE S1 — The growth curve of V82, VL8 at different temperature stages. [file Image_1.TIF]

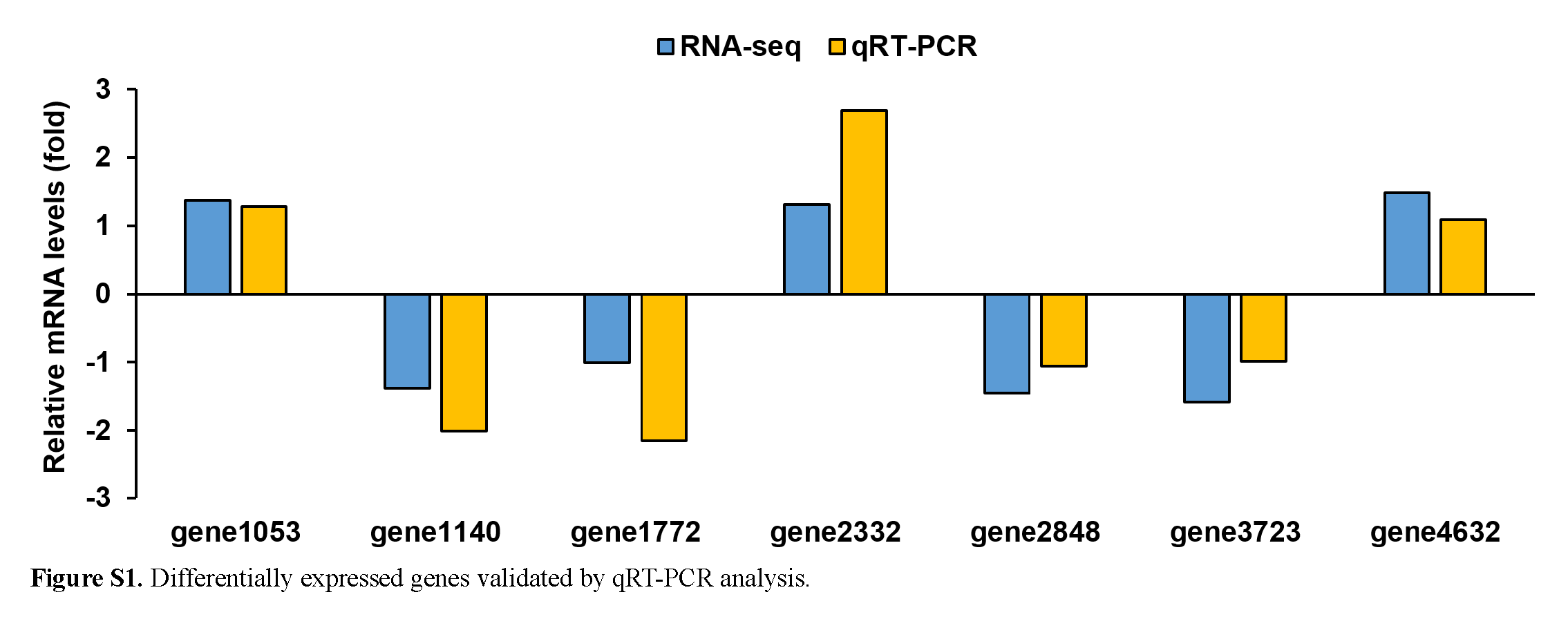

Supplement: FIGURE S2 — Differentially expressed genes validated by qRT-PCR analysis. [file Image_2.TIF]

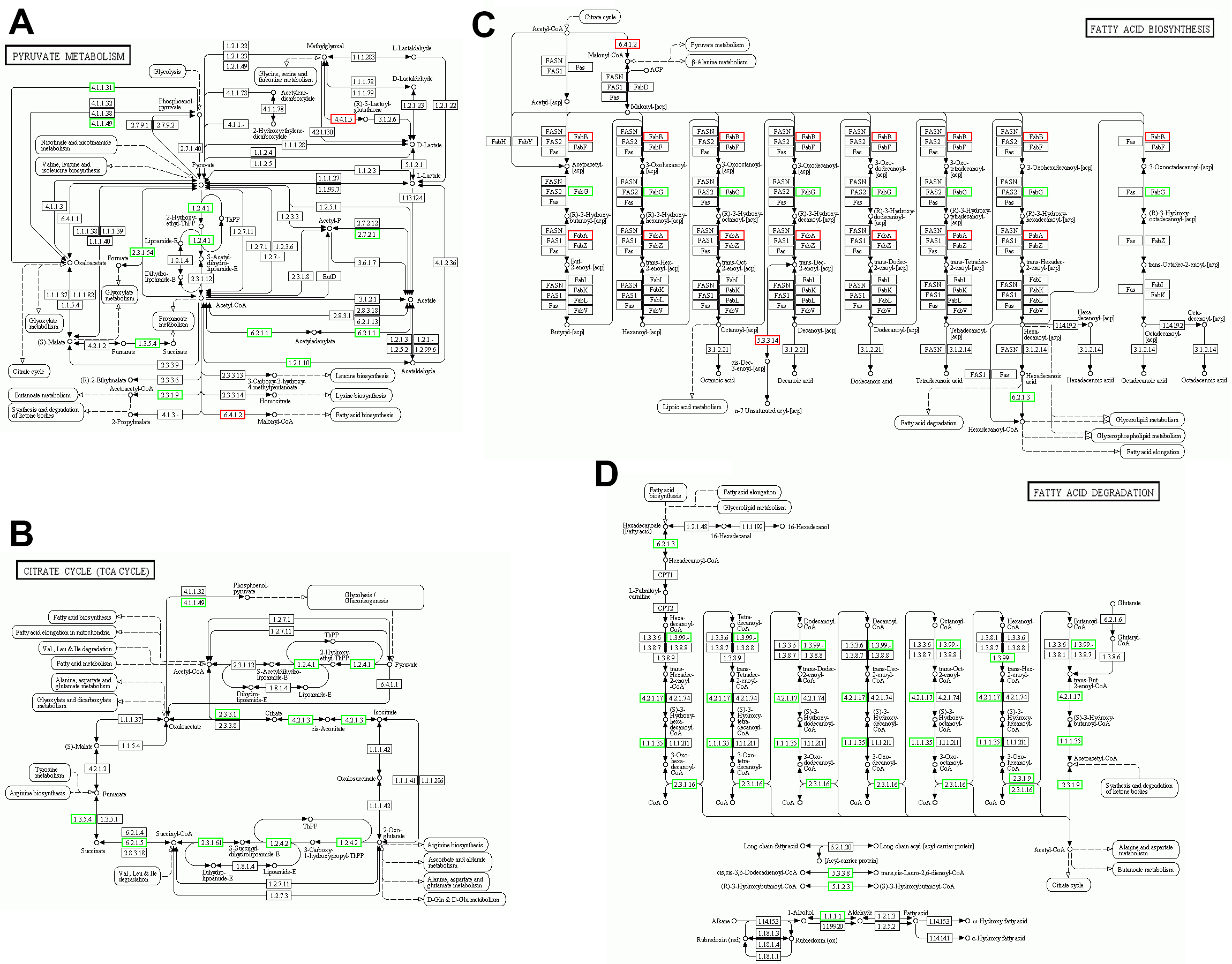

Supplement: FIGURE S3 — The important pathways affected by cold stress in V. parahaemolyticus, including pyruvate metabolism pathway (A), citrate cycle (TCA cycle) pathway (B), fatty acid biosynthesis pathway (C), and fatty acid degradation pathway (D). Red indicates that the gene is up-regulated at low temperature, while green indicates that the gene is down-regulated at low temperature. [file Image_3.TIF]

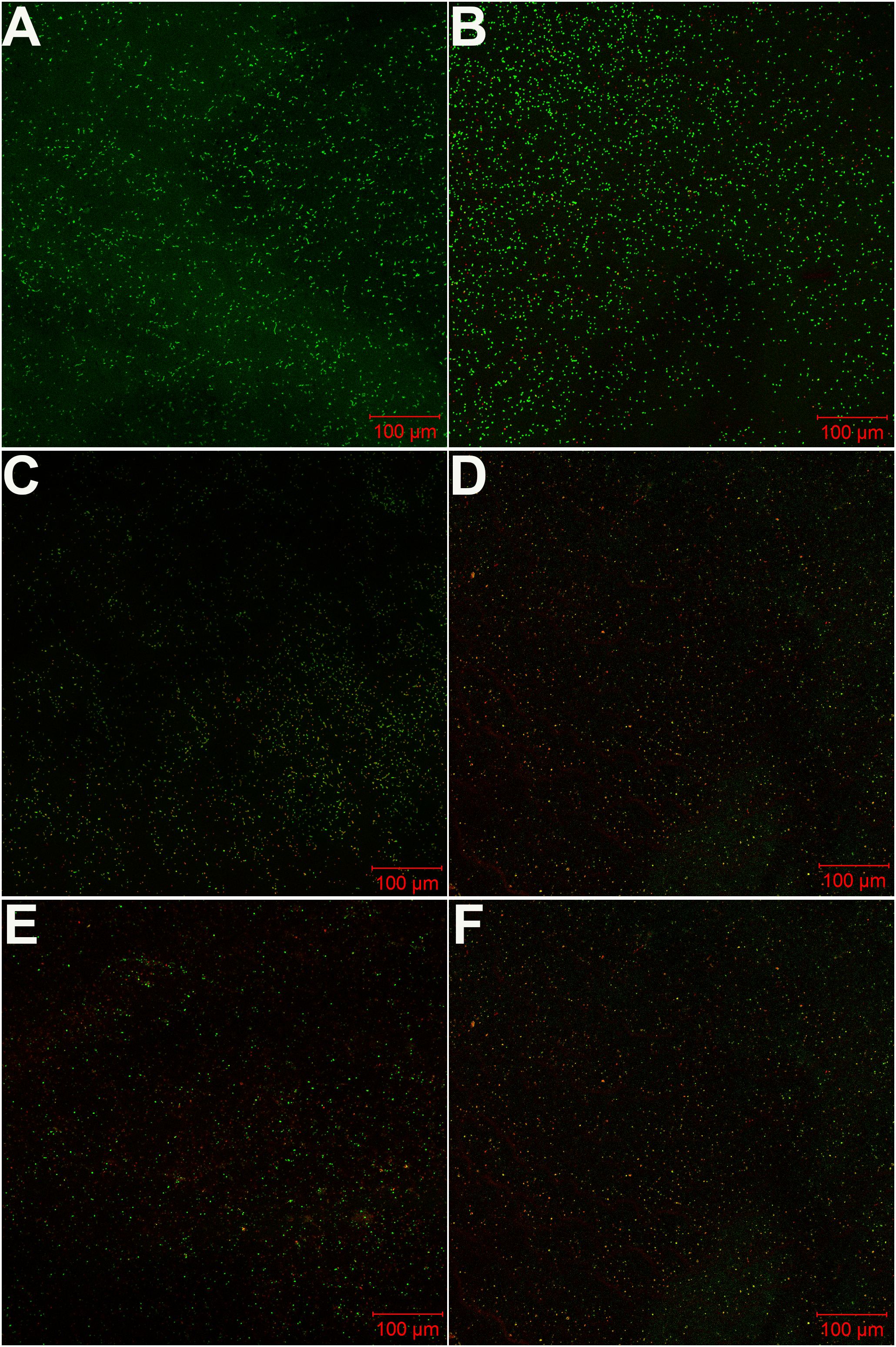

Supplement: FIGURE S4 — Laser confocal fluorescence microscopy indicating the status of bacteria supplied with different doses of pyruvate at 4°C. (A) Initial sample at 37°C without any treatment. (B) Sample at 37°C for 1 h. (C) Sample without pyruvate supplement at 4°C for 1 h. (D) Sample with 2.5 g/L pyruvate supplement at 4°C for 1 h. (E) Sample with 10 g/L pyruvate supplement at 4°C for 1 h. (F) Sample with 20 g/L pyruvate supplement at 4°C for 1 h. Green cells represent the alive bacteria, while red cells represent the dead bacteria. [file Image_4.TIF]
